# Development of Phenotyping Algorithms for the Identification of Organ Transplant Recipients: Cohort Study

**DOI:** 10.2196/18001

**Published:** 2020-12-10

**Authors:** Lee Wheless, Laura Baker, LaVar Edwards, Nimay Anand, Kelly Birdwell, Allison Hanlon, Mary-Margaret Chren

**Affiliations:** 1 Department of Dermatology Vanderbilt University Medical Center Nashville, TN United States; 2 Meharry Medical College Nashville, TN United States; 3 Division of Nephrology and Hypertension Department of Medicine Vanderbilt University Medical Center Nashville, TN United States

**Keywords:** phenotyping, electronic health record, organ transplant recipients

## Abstract

**Background:**

Studies involving organ transplant recipients (OTRs) are often limited to the variables collected in the national Scientific Registry of Transplant Recipients database. Electronic health records contain additional variables that can augment this data source if OTRs can be identified accurately.

**Objective:**

The aim of this study was to develop phenotyping algorithms to identify OTRs from electronic health records.

**Methods:**

We used Vanderbilt’s deidentified version of its electronic health record database, which contains nearly 3 million subjects, to develop algorithms to identify OTRs. We identified all 19,817 individuals with at least one International Classification of Diseases (ICD) or Current Procedural Terminology (CPT) code for organ transplantation. We performed a chart review on 1350 randomly selected individuals to determine the transplant status. We constructed machine learning models to calculate positive predictive values and sensitivity for combinations of codes by using classification and regression trees, random forest, and extreme gradient boosting algorithms.

**Results:**

Of the 1350 reviewed patient charts, 827 were organ transplant recipients while 511 had no record of a transplant, and 12 were equivocal. Most patients with only 1 or 2 transplant codes did not have a transplant. The most common reasons for being labeled a nontransplant patient were the lack of data (229/511, 44.8%) or the patient being evaluated for an organ transplant (174/511, 34.1%). All 3 machine learning algorithms identified OTRs with overall >90% positive predictive value and >88% sensitivity.

**Conclusions:**

Electronic health records linked to biobanks are increasingly used to conduct large-scale studies but have not been well-utilized in organ transplantation research. We present rigorously evaluated methods for phenotyping OTRs from electronic health records that will enable the use of the full spectrum of clinical data in transplant research. Using several different machine learning algorithms, we were able to identify transplant cases with high accuracy by using only ICD and CPT codes.

## Introduction

The Scientific Registry for Transplant Recipients (SRTR) is an outstanding resource for studies of organ transplant recipients (OTRs). The SRTR has incomplete data on important variables such as cancers in transplant patients and lacks a common data model [1-3]. Linking records to cancer registries has greatly aided in the collection of these data, but not all outcomes can be measured in this way [4]. Moreover, the regulations regarding linking these identified data sets to DNA biobanks can be burdensome and limit the scale of genetic studies that can be conducted in OTRs. To address these limitations, other resources that contain a more robust record of patients’ health, such as the electronic health record (EHR), can be used [5]. The use of different types of data contained in the EHR to phenotype disease states has gained broad acceptance [6-8]. Most studies seeking broader data have attempted to link EHR data to the SRTR [9-11]. This approach can be problematic because to protect patient privacy according to the Health Insurance Portability and Accountability Act, the linkage is done by the SRTR management team, with new identifiers returned to the investigator. These new identifiers preclude linkage back for updating or correcting records or linking to deidentified genetic databases.

To avoid this issue, several studies have used the presence of an International Classification of Diseases (ICD)-9 or ICD-10 code or Current Procedural Terminology (CPT) code for transplantation to identify transplant patients, although this practice is known to have poor performance [9-11]. ICD codes are used as a means of providing distinct diagnoses for billing purposes. ICD version 9 was first used in 1979 and it ran until October 1, 2014 in the United States, at which time ICD-10 was adopted. Patients whose records span this timepoint thus can contain both ICD-9 and ICD-10 codes in their records, whereas patients seen only prior to then would have exclusively ICD-9 codes. CPT codes designate specific surgeries and procedures. A thorough investigation of the accuracy of using ICD and CPT codes to phenotype OTRs has not been performed nor have formal phenotyping algorithms for identifying transplant patients from the EHR been developed. We therefore conducted this study to develop rigorously evaluated phenotyping algorithms for the identification of transplant patients from EHRs.

## Methods

### Cohort Assembly

This study used deidentified patient-level data and was designated as an exempt nonhuman subjects research study by the institutional review board at the Vanderbilt University Medical Center (VUMC). We identified all possible OTRs from the Synthetic Derivative [12]. The Synthetic Derivative contains over 2.9 million subjects with deidentified clinical data from the EHR collected longitudinally over several decades since VUMC began using an EHR. The Synthetic Derivative is linked to a large DNA biobank called BioVU [12]. Similar to the entire patient population seen at VUMC, patients are predominantly Caucasian, and there are approximately equal numbers of males and females. The Synthetic Derivative includes all information available in the EHR, incorporating diagnostic codes (ICD-9 and ICD-10), CPT codes, demographics, text from inpatient and outpatient notes (including both subspecialty and primary care), laboratory values, radiology reports, and medication orders. However, records scanned into the EHR are not available in the Synthetic Derivative. Users can perform text-based searches of the entire clinical record within seconds to increase the efficiency and accuracy of data extraction. To identify possible OTRs within the Synthetic Derivative, we used ICD-9 and ICD-10 codes as well as CPT codes specific to each organ (Table 1). We excluded codes for bone, cornea, and skin transplants, as these are uncommon. Although bone marrow and stem cell transplants are not included in SRTR, we included these, given the large number of transplants performed every year and the need to be able to identify these patients.

**Figure 1:**
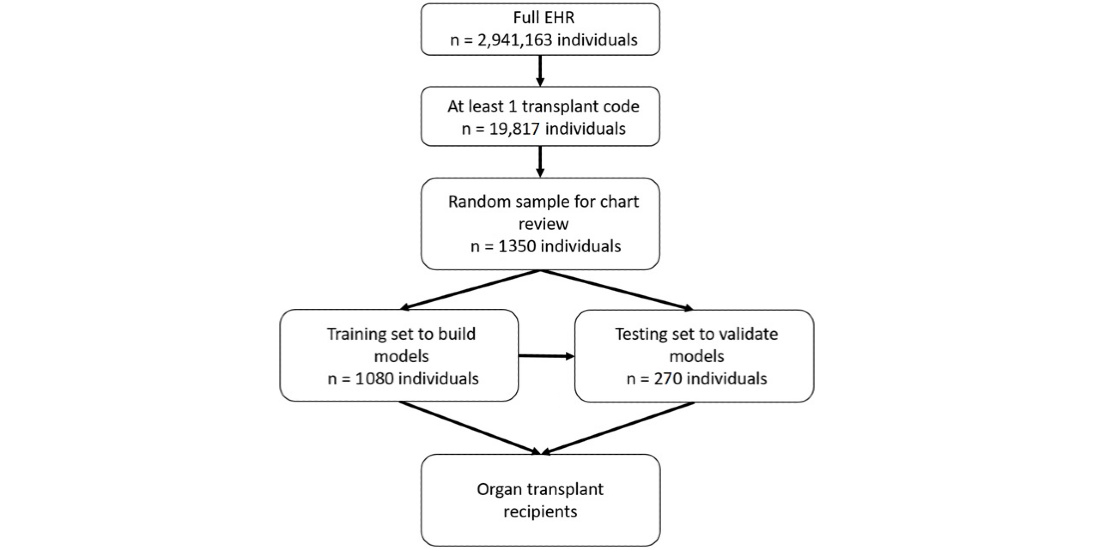
Selection of patients. From the full electronic health record, we identified 19,817 individuals with at least one transplant code, and from these, we selected a random sample of 1350 individuals for chart review and model building. EHR: electronic health record.

**Figure 2:**
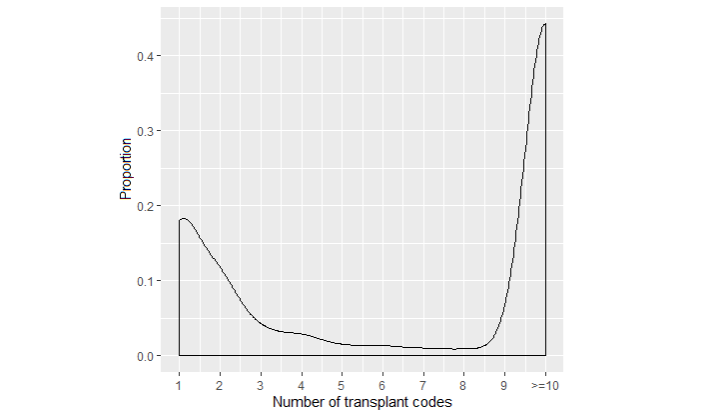
Frequencies of total transplant code counts among those 19,817 individuals with at least one transplant code.

We randomly selected 1350 patients for chart review to confirm organ transplant status and to serve as training and testing sets (Figure 1). A preliminary analysis of the first 750 charts showed difficulty in the models correctly identifying OTRs with a low number of codes. Overall, there was a bimodal distribution of code count frequencies, with high numbers of patients having only 1 or 2 and >50% having 10 or more codes (Figure 2). Therefore, we reviewed an additional 500 charts with oversampling of those with 1 or 2 codes. There were only 31 lung transplant cases included in the initial sample; therefore, we reviewed an additional 100 charts that had at least one code for lung transplant to increase the sample size. Chart review was performed by 3 authors (LW, LXW, NA) with 20% overlap to determine interrater reliability. Disagreements were settled by reviewers examining the record in question together to make a final determination. The time of possible transplant was defined as the date of the first CPT code for transplant or the earliest transplant code in the chart. Transplant patients were defined as those with any definitive evidence of having a transplant (eg, transplant procedure note, transplant biopsy pathology report, documentation in the chart of having a transplant). Equivocal cases were defined as those with an absence of definitive evidence but with factors potentially related to transplantation (eg, subsequent immunosuppressant use, laboratories measuring tacrolimus levels, multiple cytomegalovirus titers). Patients without documentation of a transplant were defined as those with definitive evidence of having not received a transplant (eg, organ donation, denied listing for transplantation). Patients whose charts contained only ICD and CPT codes but lacking any documentation of notes, pathology records, radiology records, laboratory records, or medications were classified as not having evidence of a transplant unless there were multiple transplant codes at different time points.

**Table 1:** List of the International Classification of Diseases and Current Procedural Terminology codes used to identify possible organ transplant recipients from the electronic health record.

Transplanted organ	ICD^a^-9 codes	ICD-10 codes	Current Procedural Terminology codes
Heart	V42.1, 996.83, 37.51	Z94.1, Z94.3, T86.2, T86.3, 02YA0Z^b^	33935, 33945
Lung	V42.6, 996.84	Z94.2, Z94.3, T86.3, T86.81, 0BY^b^	32851, 32852, 32853, 32854
Kidney	V42.0, 996.81	Z94.0, T86.1, 0TY^b^	50340, 50370, 50380, 50360, 50365
Liver	V42.7, 996.82	Z94.4, T86.4, 0FY00^b^	47135, 47136
Bone marrow or stem cell	V42.81, V42.82, 996.85, 996.88, 41.0, 41.00, 41.01, 41.02, 41.03, 41.04, 41.05, 41.06, 41.07, 41.08, 41.09	Z94.81, Z94.84, T86.0, T86.5, 30230A^b^, 30230G^b^, 30230X^b^, 30230Y^b^, 30233A^b^, 30233G^b^, 30233X^b^, 30233Y^b^, 30240A^b^, 30240G^b^, 30240X^b^, 30240Y^b^, 30243A^b^, 30243G^b^, 30243X^b^, 30243Y^b^, 30250G^b^, 30250X^b^, 30250Y^b^, 30253G^b^, 30253X^b^, 30253Y^b^, 30260G^b^, 30260X^b^, 30260Y^b^, 30263G^b^, 30263X^b^, 30263Y^b^	38242, 38240, 38241, 38243
Pancreas, intestine, or other	V42.83, V42.84, V42.89, V42.8, V42.83, V42.9, 996.86, 996.87, 996.89, 996.80	Z94.82, Z94.83, Z94.89, Z94.9, T86.85, T86.89, T86.90, T86.91, T86.92, T86.93, T86.99, 0FYG0Z^b^	48554, 48556

^a^International Classification of Diseases.

^b^Means all values under this subheading, eg, “0FYG0Z*” includes 0FYG0Z0, 0FYG0Z1, and 0FYG0Z2.

### Algorithm Development

We split the population of 1350 into a training set of 1080 individuals (80.0%) and a testing set of the remaining 270 individuals (20.0%). We calculated the positive predictive value (PPV), sensitivity, and F-score at each sequential cut point from each sequential cut point (>1, >2, >3…>10) of the total ICD-9, ICD-10, and CPT transplant codes, labeling those below the cut point as nontransplant patients and those above the cut point as transplant patients. We selected the cut point with the highest F-score in the training set and calculated these values in the test set by using the same cut point. We considered several different models, starting with classification and regression trees (CART), which is perhaps the most approachable to clinicians without any formal training in bioinformatics and then expanding to ensemble methods of random forest (RF) and extreme gradient boosting (XGB). The variables used in the models included age at transplant, race, gender, year of transplant, duration of follow-up, vital status, the codes listed in Table 1, total number of transplant codes, total number of transplant status codes, total number of transplant procedure codes, total number of transplant complications codes, and total number of transplant aftercare codes. Machine learning models were constructed using the training set with 5-fold cross validation and were tuned using the caret package in R 3.5.1 [13,14]. The final tuning parameters for each model are presented in Table S1 of Multimedia Appendix 1. The rpart package was used for CART models [15], the ranger package was used for RF models [16], and the xgboost package was used for XGB models by using method = “xgbTree” in the caret framework [17]. Sensitivity was defined as the number of those predicted as having a transplant divided by the total number of transplant patients. PPV was the number of transplant patients correctly predicted to have a transplant divided by the total number of patients predicted to be transplant patients. Sensitivity and PPV were calculated overall and for each organ type. All models were compared using the F-score, which is calculated as 2*(sensitivity*PPV)/(sensitivity + PPV). An F-score of 1.0 represents perfect classification. Because all charts were selected based on the presence of a transplant code, specificity could not be calculated.

### Alternative Search Strategies

Preliminary models suggested difficulty in discriminating between transplant recipients and nontransplant recipients with fewer than 4 transplant codes. We therefore considered the addition of medication and laboratory data. However, among these subjects with few codes, we found that nearly all of them had data for only ICD and CPT codes and not medications; therefore, this strategy was abandoned. We also considered the addition of natural language processing (NLP) methods to augment the search algorithms. While this 2-step process has shown better performance than using codes alone, we observed that the model had excellent performance in patients with unstructured data sources and poor performance in those without unstructured data [18]. As such, the addition of NLP would have improved our classification only minimally, while greatly increasing the complexity of the algorithm. All the algorithms were therefore constructed using the structured data only.

## Results

### Cohort Assembly

Among patients in the Synthetic Derivative with at least one transplant code, there were 7751 potential renal transplant patients, 3240 potential cardiac transplant patients, 1506 potential lung transplant patients, 3648 potential liver transplant patients, 6401 potential stem cell or bone marrow transplant patients, and 3845 patients potentially with a transplanted pancreas, small intestine, or other organs besides skin, bone, or eye. Accounting for patients with codes for multiple transplanted organs, there were 19,817 unique individuals.

The mean number of codes per individual was 52.6 and the median count was 6. Many of the individuals had only 1 (4439/19,817, 22.3%) or 2 (2243/19,817, 11.3%) transplant codes (Figure 2). A chart review of 1350 subjects revealed 827 (61.3%) transplant patients, 12 (0.9%) equivocal cases, and 511 (37.9%) patients without documentation of a transplant. Individuals with a greater number of codes were more likely to be OTRs (Table 2). Interrater reliability was extremely high (247/250, 98.8% concordance), and all 3 discrepancies involved patients being labeled as OTRs versus equivocal. The most common reasons for being labeled as not having documentation of a transplant were the lack of adequate data (229/511, 44.8%) or the patient currently or formerly being evaluated for an organ transplant (174/511, 34.1%). Other reasons included coding errors identified during the chart review, such as the patient receiving blood products or tagged red blood cell scans. In preliminary analyses, we considered models excluding the 12 equivocal cases or categorizing them as OTRs or non-OTRs. There were no material differences among the models; therefore, these 12 were labeled as cases in the final models presented.

**Table 2:** Frequencies, positive predictive value, sensitivity, and F-score by code counts of organ transplant recipients and nonorgan transplant recipients.

Transplant codes	1	2	3	4	5	6	7	8	9	10
Non-OTR^a^, n	269	173	27	12	8	8	2	1	2	9
OTR, n	51	95	24	21	8	7	6	4	16	607
PPV^b^	0.621	0.765	0.909	0.941	0.956	0.967	0.978	0.981	0.983	0.985
Sensitivity	1.000	0.939	0.879	0.797	0.772	0.763	0.754	0.747	0.743	0.723
F-score	0.767	0.843	0.894	0.863	0.854	0.853	0.852	0.848	0.846	0.834

^a^OTR: organ transplant recipient.

^b^PPV: positive predictive value.

### Models for Overall Transplant Status

Using 3 or more codes as the cut point for calling a patient a transplant recipient had the highest F-score (Table 2). The sensitivity and PPV of the code counts and the CART, RF, and XGB models for identifying OTRs are shown in Table 3. CART, RF, and XGB all performed comparably, with RF having the highest F-score in the testing set. Applying the overall RF model to the full study population yielded a final sample size of 13,445 OTRs. For comparison, VUMC has performed 7671 solid organ transplants between January 1, 1988 and February 28, 2019, and 1323 bone marrow and stem cell transplants from 2015 to 2018 [19,20].

**Table 3:** Positive predictive value, sensitivity, and F-scores for each model to identify individuals with any organ transplant in the training and testing sets.

Model	Training set			Testing set		
	PPV^a^	Sensitivity	F-score	PPV	Sensitivity	F-score
>3 codes	0.909	0.876	0.892	0.911	0.911	0.911
CART^b^	0.911	0.872	0.891	0.903	0.892	0.898
RF^c^	0.909	0.887	0.898	0.909	0.909	0.909
XGB^d^	0.925	0.882	0.903	0.846	0.892	0.868

^a^PPV: positive predictive value.

^b^CART: classification and regression tree.

^c^RF: random forest.

^d^XGB: extreme gradient boosting.

### Organ-Specific Models

Many patients had codes for >1 organ type; therefore, we included all of the codes in organ-specific models. The 2 most important variables in these models in all 3 algorithms included codes for either the correct organ transplant status (V42 and Z94 codes, with decimals specifying organ type), complications of the correct transplanted organ (996 or T86 codes, with decimals specifying organ type), or procedural codes specifying the correct organ type (Table S2 of Multimedia Appendix 1). The PPV, sensitivity, and F-scores for the training and testing sets for each organ type are presented in Table 4.

**Table 4:** Positive predictive value, sensitivity, and F-score for each machine learning model to identify individuals with specific organ transplant types in the training and testing sets.

Organ, model		Training set			Testing set		
		PPV^a^	Sensitivity	F-score	PPV	Sensitivity	F-score
**Heart**							
	>5 codes	0.974	0.8	0.879	1	0.8	0.889
	CART^b^	0.94	0.732	0.824	0.75	0.923	0.828
	RF^c^	0.972	0.814	0.886	0.875	1	0.933
	XGB^d^	0.972	0.814	0.886	0.875	0.875	0.875
**Lung**							
	>4 codes	0.919	0.872	0.895	1	1	1
	CART	0.868	0.78	0.821	0.864	1	0.927
	RF	1	0.915	0.956	0.864	1	0.927
	XGB	0.981	0.898	0.938	0.864	1	0.927
**Kidney**							
	>4 codes	0.918	0.789	0.849	0.947	0.818	0.878
	CART	0.824	0.84	0.832	0.887	0.94	0.913
	RF	0.901	0.84	0.869	0.943	0.893	0.917
	XGB	0.888	0.85	0.868	0.906	0.906	0.906
**Liver**							
	>6 codes	0.963	0.89	0.925	1	1	1
	CART	0.928	0.865	0.896	0.95	0.792	0.864
	RF	0.979	0.894	0.935	1	1	1
	XGB	0.979	0.904	0.94	1	0.952	0.976
**Bone marrow**							
	>6 codes	0.918	0.69	0.788	0.933	0.875	0.903
	CART	0.862	0.884	0.873	0.882	0.789	0.833
	RF	0.932	0.828	0.877	0.863	0.898	0.88
	XGB	0.909	0.859	0.883	0.863	0.846	0.854

^a^PPV: positive predictive value.

^b^CART: classification and regression tree.

^c^RF: random forest.

^d^XGB: extreme gradient boosting.

### Sensitivity Analyses

The United States transitioned from ICD-9 to ICD-10 coding on October 1, 2014. We examined if the model performance differed before or after this time point and found good stability overall. For example, the XGB model for overall transplant status had an F-score of 0.92 before and 0.89 after October 1, 2014. We also noted that the majority of our cases underwent a transplant after the year 2000. We examined model performance before and after January 1, 2000 and found very stable F-scores (0.91 before and 0.92 after in the XGB model for overall transplant status), suggesting little impact on the model based on this imbalance.

## Discussion

In this study, we developed and validated phenotyping algorithms for identifying OTRs from the EHR. Using several different rule-based and machine learning methods, we were able to identify OTRs overall with 90% PPV and sensitivity and higher values for several individual organ types. The algorithms all performed comparably well, although RF tended to be the most consistent. The development of these phenotyping algorithms was necessary as the PPV for using at least one transplant code to identify OTRs was only 60%, indicating that studies based on the presence of only one of these codes may have biased results.

The SRTR of the United Network for Organ Sharing and the Organ Procurement and Transplant Network is the primary national database for transplant recipient outcomes research. Because the SRTR is not linked directly to patient records in EHRs, it is not possible to collect data on additional variables not captured by the data entry forms. As a result, many important variables and outcomes are completely omitted. Indeed, a recent study of cardiac transplants using SRTR data found that advanced machine learning methods did not outperform the more traditional prediction models for 1-year survival, with the authors concluding that the methods were hindered by limited data in the registry [21]. By developing validated algorithms to identify OTRs from the EHR, a broader range of studies can be conducted using the data in the full clinical record.

Large reviews of the accuracy of diagnostic and procedural codes show <90% concordance with true diagnoses in inpatient and outpatient settings, both in the United States and other countries [9,22,23]. In a study from Canada, the use of ICD codes alone to identify kidney donors had only 60% sensitivity and 78% PPV, which were similar to our findings for transplant recipients [9]. While the primary diagnosis for a visit is less likely to be missed, secondary diagnoses were more likely to be omitted from the coding. In the United States, up to 12 diagnoses can be entered for an encounter, though only 4 are allowed to be linked to an individual service, with the codes generating the highest reimbursements being prioritized by the medical coders. As a result, transplant patients seen for critical illnesses or procedures may have been less likely to have a transplant code listed.

Many of the charts we reviewed contained only 1 or 2 transplant codes. In addition, these charts often had only ICD and CPT codes but no documents, medications, or laboratory data. Two possible explanations for this lack of data are that handwritten notes and outside records are not scanned into the Synthetic Derivative, and patients with sparse data that could make them potentially identifiable are redacted more often than those with deeper coverage of their records. Regardless of the reason for lack of data, these patients were all called nontransplant patients, and therefore, our algorithm might underestimate the PPV for those with few codes. We attempted to improve our accuracy in classifying these individuals with few transplant codes. First, after identifying this problem in our preliminary analyses, we increased our initial sample by 67% with oversampling of those with only 1 or 2 codes to provide the models with more data points with which to learn to classify them. We also considered adding medications to our algorithms as well as applying NLP to the documents in the EHR. Although these strategies might have augmented the PPV and sensitivity, the gains would have been minimal as those individuals with data besides ICD and CPT codes tended to have a higher number of transplant codes, and therefore, the algorithms had more accurate classification of these patients without the extra data. Moreover, classifying individuals with sparse data as non-OTRs eliminates even those true OTRs who would be excluded from later analyses due to missing data. The true transplant cases that were misclassified were almost exclusively those who had only a single presentation to VUMC with no additional follow-up. Thus, they tended to have only 1 or 2 diagnostic or procedural codes. From a broader standpoint, these were patients who also had little data to contribute to any downstream analyses of the cohort. Therefore, while the models excluded some cases, the overall information loss was low.

There was notable variation in the model metrics both within and between organ types. The reason for the different performance was likely 2-fold. First, there were low numbers for lung transplant recipients (n=81) compared to kidney transplant recipients (n=259); therefore, it is not surprising that the kidney models performed better. Second, the number of different codes contributing to a specific organ type also played a role. For example, although there were 249 stem cell or bone marrow transplant patients, there were 50 different ICD and CPT codes for this type of transplant. Therefore, it is not surprising that the bone marrow models tended to perform worse than the other organ types that had far fewer codes associated, as there were likely subsets within the cross-validations that did not include certain codes. Each code is used in different clinical settings and can be subject to individual coding preferences; therefore, this variability would be expected across institutions.

This study had several limitations. All the data were from a single medical center and coding practices may differ among institutions. Any center wishing to use this approach would need to perform a validation step to confirm the models’ performance, although EHR algorithms have been shown to have good portability between populations [24]. VUMC is a high-volume transplant center, and as a result, many patients are seen there for either transplant surgery alone or for follow-up after receiving a transplant elsewhere. This fragmentation of care can limit the available data. Our models consistently predicted slightly greater numbers of OTRs than the number of transplant procedures that have been performed at VUMC. These numbers suggest that we are in fact correctly labeling the majority of those transplants performed at VUMC, while also capturing those whose transplants were performed elsewhere but have been seen in follow-up at VUMC. More than half of the possible OTRs in our EHR had >10 transplant codes, indicating high-density data for these individuals. If we had used >10 transplant codes as our cutoff for OTR determination, the PPV would be 98.5% and the sensitivity would still be 72.3%. Conversely, a large proportion of our cohort had low numbers of transplant codes, which can correlate with the duration of the follow-up. Although the cases identified with low numbers of codes could have easily been excluded a priori by requiring a set number of total codes, doing so would falsely inflate our sensitivity measures, as many true cases would not have been investigated and confirmed on chart review. Our goal was to provide accurate estimates of the algorithm’s overall performance, even if many of the identified cases would ultimately be excluded due to missing data in subsequent analyses. Many patients had no available text data from notes. This deficiency likely was the outcome of handwritten notes not being included in the Synthetic Derivative. Thus, we were not able to add NLP to our algorithms, which potentially could have improved our models. EHRs can be a powerful tool for investigating outcomes not captured by large registries.

In this study, we have validated algorithms for identifying OTR overall and OTRs receiving specific organs by using only ICD and CPT codes. Single variable phenotyping algorithms based on code counts alone perform well but can be improved by using RFs. These algorithms can be used to construct EHR-based cohorts to broaden the range of clinical and translational studies conducted on organ transplants.

## Appendix

Multimedia Appendix 1 Tuning parameters and variable importance for final models.

## Abbreviations

CART: classification and regression tree
CPT: current procedural terminology
EHR: electronic health record
ICD: International Classification of Diseases
NLP: natural language processing
OTR: organ transplant recipient
PPV: positive predictive value
RF: random forest
SRTR: scientific registry for transplant recipients
VUMC: Vanderbilt University Medical Center
XGB: extreme gradient boosting
